# Clinical Characteristics and Determinants of Healing in Older Adults with Chronic Lower Extremity Ulcers: A Retrospective Cohort Study

**DOI:** 10.3390/jcm15156030

**Published:** 2026-08-03

**Authors:** Berkay Temel, Hale Nur Ertugay Aral, Esra Adışen, Ahmet Burhan Aksakal

**Affiliations:** Department of Dermatology, Faculty of Medicine, Gazi University, Emniyet Mahallesi, Mevlana Bulvarı, No:29, Ankara 06560, Turkey; halertugay@gmail.com (H.N.E.A.); adisenesra@gmail.com (E.A.); ahmetburhanaksakal@gmail.com (A.B.A.)

**Keywords:** chronic lower extremity ulcers, older adults, wound healing, wound infection

## Abstract

**Background/Objectives:** Chronic lower extremity ulcers are a major cause of morbidity in older adults. However, real-world data on clinical characteristics, microbiological findings, treatment patterns, and outcomes in older adults remain limited. The objective of this study was to evaluate the clinical characteristics and factors associated with healing in older adult patients with chronic lower extremity ulcers. **Methods:** This retrospective cohort study included patients aged ≥65 years with chronic lower extremity ulcers treated at a tertiary dermatology center between January 2016 and March 2025. Demographic, clinical, microbiological, treatment, and outcome data were collected from medical records. Factors associated with healing outcomes were evaluated using regression analyses. **Results:** A total of 191 patients were included. Venous ulcers were the most common ulcer subtype (37.2%), followed by diabetic ulcers (19.4%). Wound cultures were positive in 48.2% of patients, with *Staphylococcus aureus* being the most frequently isolated organism. Complete healing was achieved in 18.8% of patients, while 29.6% experienced partial healing. Peripheral arterial disease (adjusted OR 0.25, 95% CI 0.09–0.64) and greater ulcer depth (adjusted OR 0.45, 95% CI 0.20–0.91) were independently associated with a lower likelihood of complete healing. Greater ulcer depth and positive wound cultures were independently associated with non-healing. Peripheral arterial disease and greater ulcer depth were also associated with delayed healing. **Conclusions:** Chronic lower extremity ulcers in older adults are associated with poor healing outcomes. Peripheral arterial disease, ulcer depth, and wound infection emerged as key determinants of healing and may help identify patients requiring closer monitoring and multidisciplinary management.

## 1. Introduction

Chronic lower extremity ulcers represent a significant challenge in clinical practice, particularly among older adults. As life expectancy increases and chronic diseases become more prevalent, the burden of non-healing wounds in geriatric populations continues to grow. These wounds are associated with prolonged treatment, recurrent infections, pain, impaired mobility, reduced quality of life, and increased healthcare utilization [1,2].

Wound healing is a complex and dynamic process involving inflammatory, proliferative, and remodeling phases. Aging negatively affects many components of this process, including collagen synthesis, angiogenesis, fibroblast function, and immune responses. These changes may delay tissue repair and increase susceptibility to wound chronicity [3,4]. Older patients are also more likely to have comorbid conditions such as diabetes mellitus, chronic venous insufficiency, peripheral arterial disease, cardiovascular disease, and chronic kidney disease. These conditions may impair wound healing and complicate clinical management [5].

The clinical presentation of chronic lower extremity ulcers in older adults is highly heterogeneous. Venous, arterial, and diabetic ulcers are among the most frequently encountered types [5]. However, dermatologists also frequently encounter ulcers associated with vasculitis, inflammatory dermatoses, medications, malignancies, pressure injury, and lymphatic disorders, and multiple etiologic factors may coexist in the same patient. As a result, the spectrum of chronic lower extremity ulcers seen in routine dermatology practice is often broader than that represented in studies focusing on a single ulcer subtype.

Microbial colonization and infection are additional factors that may influence wound progression and healing. Chronic wounds frequently harbor multiple microorganisms, including biofilm-forming bacteria. Biofilms can maintain persistent inflammation, reduce treatment efficacy, and delay wound healing [6,7]. Despite their clinical importance, microbiological findings are not always evaluated together with ulcer characteristics, treatment patterns, and healing outcomes. Data addressing all of these aspects within a single geriatric cohort remain limited.

Most previous studies have focused on specific ulcer subtypes, particularly diabetic foot ulcers and venous ulcers. Consequently, the overall clinical spectrum of chronic lower extremity ulcers in older adults has not been fully characterized. A better understanding of demographic characteristics, ulcer features, microbiological findings, and factors associated with healing may help clinicians identify high-risk patients and optimize treatment strategies [8]. In this study, we aimed to describe the demographic and clinical characteristics, microbiological profile, treatment modalities, and healing outcomes of older adults with chronic lower extremity ulcers treated at a tertiary dermatology center. We also sought to identify factors independently associated with complete healing and non-healing outcomes.

## 2. Material and Methods

This retrospective cohort study was conducted at the Department of Dermatology, Gazi University Faculty of Medicine, Türkiye. Medical records of geriatric patients diagnosed with chronic lower extremity ulcers between January 2016 and March 2025 were reviewed.

**Figure 1:**
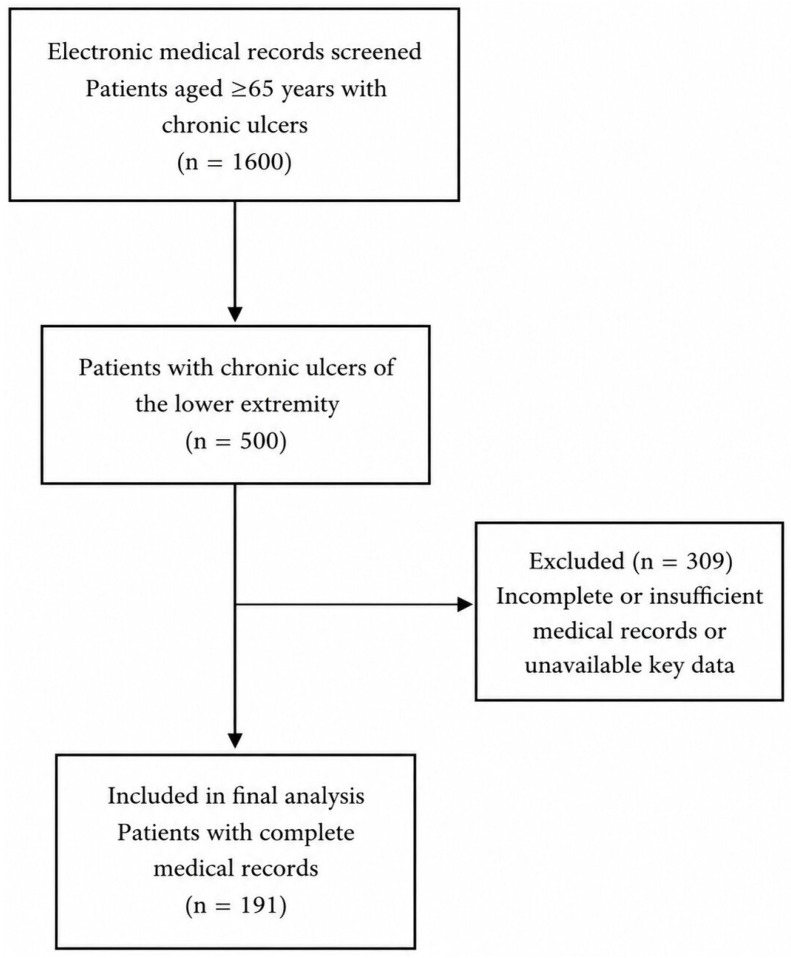
Flow diagram of patient selection.

Patients aged 65 years and older with at least one chronic lower extremity ulcer were eligible for inclusion. A chronic ulcer was defined as a skin defect of the lower extremity persisting for more than six weeks despite appropriate wound care. Patients with incomplete or insufficient clinical records were excluded. The patient selection process is presented in Figure 1.

The study was approved by the Gazi University Rectorate Clinical Research Ethics Committee (Approval No. 2025-2219) and was conducted in accordance with the Declaration of Helsinki.

Demographic and clinical data were collected from electronic medical records. Recorded variables included age, sex, body mass index (BMI), smoking status, comorbidities, medication history, and laboratory findings including C-reactive protein (CRP) and glycated hemoglobin (HbA1c). Comorbidities included diabetes mellitus, hypertension, coronary artery disease, heart failure, peripheral arterial disease, chronic kidney disease, malignancy, immunosuppression, and atrial fibrillation.

Ulcer-related variables included ulcer type, duration, anatomical location, number of ulcers, ulcer area, ulcer depth, presence of necrosis, fibrin, pain, odor, and exudate level. Ulcers were classified as venous, arterial, diabetic, vasculitic, drug-related, pyoderma gangrenosum-related, lymphatic, neoplastic, mixed (ulcers with more than one underlying etiology), or unclassified according to clinical, histopathological, microbiological, and radiological findings documented in patient records. Ulcer area was estimated from the recorded wound dimensions and was expressed in square centimeters (cm^2^). Ulcer depth was categorized as superficial, subcutaneous, muscle/tendon, or bone involvement.

Results of wound surface cultures, deep tissue cultures, and blood cultures were recorded when available. Isolated microorganisms and the presence of polymicrobial growth were documented. Culture positivity was defined as any bacterial growth detected in wound swab or tissue culture. Radiological assessments, including arterial Doppler ultrasonography, venous Doppler ultrasonography, and magnetic resonance imaging findings suggestive of osteomyelitis, were also reviewed.

Treatment-related variables included topical wound care, negative-pressure wound therapy, compression therapy, debridement, platelet-rich fibrin application, topical and systemic antimicrobial treatments, revascularization procedures, skin grafting or flap surgery, and amputation.

Clinical outcomes were categorized as complete healing, partial healing, no healing, or loss to follow-up according to the last documented clinical assessment. Complete healing was defined as complete epithelialization of the ulcer. Partial healing was defined as a documented reduction in ulcer size or clinical improvement without complete closure. Recurrence was defined as reappearance of ulceration after documented healing. Healing time, recurrence, hospitalization status, and duration of hospitalization were also recorded. Patients were followed according to routine clinical practice rather than a predefined study protocol. Follow-up duration therefore varied between patients, and clinical outcomes were determined based on the last documented follow-up visit.

Statistical analyses were performed using R software version 4.5.1 (R Foundation for Statistical Computing, Vienna, Austria). Continuous variables were assessed for normality using the Shapiro–Wilk test and are presented as mean ± standard deviation or median (interquartile range [IQR]) as appropriate. Categorical variables are summarized as frequencies and percentages. Baseline characteristics were compared between patients with documented follow-up outcomes and those lost to follow-up. Continuous variables were compared using the Mann–Whitney U test, whereas categorical variables were compared using the chi-square test or Fisher’s exact test, as appropriate.

Factors associated with complete healing, non-healing, and time to complete healing were evaluated using regression analyses. All clinically relevant candidate variables were initially evaluated in univariable analyses. Variables were not selected for multivariable modeling solely on the basis of univariable statistical significance. Given the limited number of outcome events, particularly the 35 complete healing events, the number of predictors was restricted to minimize overfitting. Predictors were selected a priori based on clinical relevance and biological plausibility while considering the number of events available for each outcome. Multivariable models were constructed using Firth penalized logistic regression. Time to complete healing was analyzed using Kaplan–Meier survival analysis and Cox proportional hazards regression. All regression analyses were performed at the patient level and were not stratified by ulcer etiology because of the small number of patients in several ulcer subgroups. Ulcer depth was entered into the regression models as an ordinal variable (superficial, subcutaneous tissue, muscle/tendon, and bone involvement), with each one-grade increase representing greater ulcer depth. Complete-case analyses were performed for each regression model, with patients excluded only if data were missing for variables included in the respective model. The proportional hazards assumption was assessed using Schoenfeld residuals, and restricted mean survival time analyses were performed when appropriate. A two-sided *p* value < 0.05 was considered statistically significant.

## 3. Results

A total of 191 older patients with chronic lower extremity ulcers were included in the study. The median age was 70 years (IQR, 64–76), and 111 patients (58.1%) were male. Diabetes mellitus and hypertension were the most common comorbidities, affecting 59.7% and 56.5% of patients, respectively. Peripheral arterial disease was present in 28.3% of patients, while chronic kidney disease and malignancy were observed in 22.0% and 18.8% of patients, respectively. Patients had a substantial comorbidity burden, with a mean of 2.5 ± 1.6 comorbid conditions per patient (Table 1).

Venous ulcers were the most common ulcer subtype (37.2%), followed by diabetic ulcers (19.4%) and other or unclassified ulcers (13.6%). The tibial region was the most frequently affected anatomical site (45.0%). Median ulcer duration was 3 months (IQR, 1–8), and median ulcer area was 4 cm^2^ (IQR, 1–9). Most ulcers were superficial (60.7%) or extended into the subcutaneous tissue (33.0%), whereas muscle/tendon and bone involvement were uncommon. Necrosis, pain, and malodor were present in 53.2%, 68.9%, and 38.2% of patients, respectively (Table 1).

Wound swab cultures were positive in 48.2% of patients, while deep tissue and blood cultures were positive in 7.3% and 4.7%, respectively. Among positive wound cultures, *Staphylococcus aureus* was the most frequently isolated organism (34.8%), followed by *Pseudomonas* spp. (23.9%) and *Escherichia coli* (16.3%). Gram-negative organisms predominated, accounting for 63.0% of positive wound cultures, and polymicrobial growth was observed in 34.8% of cases. Abnormal arterial and venous Doppler findings were detected in 23.6% and 18.8% of patients, respectively, while MRI-confirmed osteomyelitis was present in 11.6% (Table 2).

Topical antibiotics were the most frequently used treatment modality (77.1%), followed by negative-pressure wound therapy (74.3%) and systemic antibiotics (65.4%). The median duration of systemic antibiotic therapy was 14 days (IQR, 10–28). Compression therapy and debridement were performed in 22.0% and 24.1% of patients, respectively. Revascularization procedures were required in 5.8% of cases, whereas amputation was performed in 4.7% of patients. Treatment patterns differed significantly according to ulcer type, particularly for compression therapy, immunomodulatory treatment, negative-pressure wound therapy, and amputation (all *p* < 0.01).

Outcome data were available for 186 patients. Complete healing was achieved in 35 patients (18.8%), while 55 patients (29.6%) experienced partial healing. Twenty-eight patients (15.1%) showed no evidence of healing, and 68 patients (36.6%) were lost to follow-up. Among evaluable patients excluding those lost to follow-up (*n* = 118), the complete healing rate increased to 29.7%. The median time to healing among patients with documented healing outcomes was 2 months (IQR, 1–6). During follow-up, recurrence occurred in 28 of 90 patients (31.1%) who achieved complete or partial healing, with a median time to first recurrence of 8 months (IQR, 2–30). Overall, 67 patients (35.1%) required at least one hospitalization, and the median duration of hospital stay was 20 days (IQR, 11.2–48.8) (Table 3).

Baseline characteristics were compared between patients with available outcome data and those lost to follow-up. No significant difference in age was observed between the groups (*p* = 0.947). However, patients lost to follow-up had a lower comorbidity burden (*p* = 0.045), a lower prevalence of peripheral arterial disease (*p* < 0.001), and more superficial ulcers (*p* = 0.014).

In multivariable Firth logistic regression analysis, peripheral arterial disease (adjusted OR 0.25, 95% CI 0.09–0.64, *p* = 0.003) and greater ulcer depth (adjusted OR 0.45, 95% CI 0.20–0.91, *p* = 0.026) were independently associated with a lower likelihood of complete healing. Positive wound culture was not significantly associated with complete healing (adjusted OR 0.62, 95% CI 0.26–1.44, *p* = 0.264).

In contrast, greater ulcer depth (adjusted OR 2.36, 95% CI 1.26–4.54, *p* = 0.007) and positive wound culture (adjusted OR 5.12, 95% CI 1.82–17.63, *p* = 0.001) were independently associated with non-healing outcomes.

The complete results of the univariable analyses for complete healing, non-healing, and time to complete healing are provided in Supplementary Table S1. In the multivariable Cox regression model, peripheral arterial disease (adjusted HR 0.32, 95% CI 0.13–0.79, *p* = 0.013) and greater ulcer depth (adjusted HR 0.49, 95% CI 0.25–0.93, *p* = 0.030) were associated with delayed healing. Positive wound culture was not significantly associated with time to healing (adjusted HR 0.68, 95% CI 0.34–1.34, *p* = 0.261). In a sensitivity analysis using restricted mean survival time (RMST), patients with peripheral arterial disease experienced a 1.65-month longer time to healing compared with those without peripheral arterial disease (95% CI 0.14–3.16, *p* = 0.032) (Table 4).

## 4. Discussion

The present study provides a comprehensive overview of chronic lower extremity ulcers encountered in a tertiary dermatology clinic serving older adults. Beyond describing the clinical spectrum of disease, our findings indicate that vascular status, ulcer depth, and microbiological burden are important determinants of healing outcomes in this population. Collectively, these results emphasize the multifactorial nature of wound healing and the need for individualized management strategies in geriatric patients.

Venous ulcers represented the most common ulcer subtype in our study, accounting for more than one-third of all cases, followed by diabetic and arterial ulcers. This finding is in line with previous reports showing that venous disease remains the leading cause of lower extremity ulceration in older adults [5,8,9]. At the same time, our study included a wide range of ulcer etiologies, including vasculitic, drug-related, neoplastic, lymphatic, and mixed ulcers. This heterogeneity is clinically important, because lower extremity ulcers with different etiologies may require substantially different diagnostic and therapeutic approaches. In older patients, overlapping vascular, metabolic, inflammatory, and malignant conditions may further obscure the underlying cause. Therefore, careful etiological assessment remains essential before treatment planning in geriatric patients with lower extremity ulcers [2,5].

The microbiological findings of our study further highlight the complexity of chronic wound management in older adults. Nearly half of the patients had a positive wound culture, and Gram-negative organisms predominated among isolated pathogens. Although *Staphylococcus aureus* was the most frequently identified microorganism, a substantial proportion of wounds demonstrated polymicrobial growth. These findings are consistent with previous reports showing that chronic lower extremity ulcers often harbor diverse microbial communities rather than a single causative pathogen [10,11]. The impact of microbial burden may be amplified in older adults, as age-related alterations in tissue repair and the high prevalence of comorbidities can impair wound healing and promote wound chronicity [3,12]. Chronic wounds also provide a favorable environment for biofilm formation, which can sustain inflammation, impair tissue repair, and reduce responsiveness to treatment [12]. In our study, positive wound cultures were independently associated with non-healing, supporting the concept that microbial burden remains an important determinant of wound outcomes in older adults. While differentiating colonization from true infection remains challenging in routine clinical practice, our findings suggest that microbiological assessment may provide valuable prognostic information, particularly in wounds that fail to progress despite appropriate care. In addition, underlying osteomyelitis should be considered in patients with chronic ulcers that fail to heal despite appropriate wound care. Persistent bone infection may perpetuate inflammation, impair tissue repair, and contribute to wound chronicity, particularly in older adults with multiple comorbidities [13]. Interestingly, although positive wound culture was independently associated with non-healing, it was not associated with complete healing or time to healing. One possible explanation is that wound culture positivity alone does not reliably distinguish bacterial colonization from clinically significant infection in chronic wounds. In addition, appropriate antimicrobial therapy following microbiological assessment may have mitigated the impact of positive cultures on subsequent healing. Finally, complete healing is influenced by multiple interacting factors, including tissue perfusion, ulcer depth, and patient comorbidities, which may outweigh the independent contribution of wound microbiology. Therefore, positive wound culture may be more useful for identifying patients at risk of persistent non-healing than for predicting complete healing or the duration of healing.

The treatment patterns observed in our study reflect the complexity of managing chronic lower extremity ulcers in routine clinical practice. Negative-pressure wound therapy, topical antibiotics, and systemic antibiotics were among the most frequently used interventions, indicating that many patients required advanced wound care and infection control measures. Treatment approaches varied considerably according to ulcer etiology, with compression therapy being predominantly used for venous ulcers and revascularization procedures reserved for selected patients with arterial disease. These findings underscore the heterogeneous nature of chronic lower extremity ulcers and highlight the need for individualized treatment strategies tailored to the underlying cause and wound characteristics. In addition, emerging biomaterial-based therapies, including extracellular matrix-inspired hydrogels, have shown promising preclinical results in promoting wound healing, particularly in diabetic ulcers. However, further clinical studies are needed before these approaches can be incorporated into routine clinical practice [14].

Among the factors associated with healing outcomes, peripheral arterial disease emerged as one of the strongest predictors of poor prognosis. Patients with peripheral arterial disease were less likely to achieve complete healing and experienced longer healing times than those without arterial involvement. This finding is biologically plausible, as adequate tissue perfusion is essential for oxygen delivery, inflammatory cell recruitment, collagen synthesis, and angiogenesis during wound repair [3]. Moreover, age-related endothelial dysfunction and impaired angiogenic responses may further exacerbate the adverse effects of arterial insufficiency in older adults [15,16]. Our findings are consistent with previous studies demonstrating delayed healing in patients with impaired arterial perfusion and support current vascular guidelines emphasizing the importance of early recognition and management of peripheral arterial disease in patients with chronic lower extremity ulcers [17,18]. Notably, the association remained significant across both logistic and survival analyses, underscoring the substantial impact of arterial insufficiency on wound healing in this population.

Ulcer depth was another independent predictor of unfavorable outcomes in our study. Greater ulcer depth was associated with a lower likelihood of complete healing, a higher risk of non-healing, and a longer time to healing. This association is clinically plausible, as deeper ulcers reflect more extensive tissue damage and often indicate prolonged inflammation, impaired tissue perfusion, and a greater susceptibility to infection. Previous studies have similarly identified wound-related characteristics, including ulcer size, duration, and depth, as important determinants of healing outcomes in patients with chronic lower extremity ulcers [17,19]. The prognostic significance of ulcer depth may be particularly relevant in older adults, who often have reduced regenerative capacity and multiple comorbid conditions that can further compromise tissue repair. Taken together, these findings suggest that ulcer depth is not only a marker of wound severity but also a practical indicator of healing potential that may help identify patients who require closer follow-up and more intensive management.

The overall healing outcomes observed in our study reflect the substantial clinical challenges associated with managing chronic lower extremity ulcers in older adults. Complete healing was achieved in fewer than one-fifth of all patients, although this proportion increased to nearly one-third after excluding patients lost to follow-up. While direct comparisons across studies are difficult because of differences in patient populations, ulcer etiologies, and outcome definitions, healing rates in older adults are generally lower than those reported in studies focusing on younger or more homogeneous patient populations. The relatively modest healing rates observed in our study likely reflect the combined effects of multimorbidity, vascular disease, chronic inflammation, and microbial burden, all of which were highly prevalent in our study. In addition, more than one-third of patients were lost to follow-up. This finding may itself reflect the complexity of caring for older adults with chronic wounds, who frequently have mobility limitations, require multidisciplinary care, and may continue treatment in different healthcare settings. Taken together, these findings emphasize that chronic lower extremity ulcers in older adult patients represent a long-term management challenge rather than an isolated dermatologic condition and highlight the need for coordinated, multidisciplinary approaches to improve outcomes.

A substantial proportion of patients were lost to follow-up, which may have introduced selection bias. Baseline comparisons showed that patients lost to follow-up had a lower comorbidity burden, a lower prevalence of peripheral arterial disease, and lower ulcer depth than those with available outcome data. These findings suggest that patients lost to follow-up may have had a more favorable prognosis. Therefore, the observed healing outcomes should be interpreted with caution, as the overall healing rate may have been underestimated.

## 5. Limitations

This study has several limitations. First, this was a single-center study conducted in a tertiary dermatology referral center, which may limit the generalizability of the findings to other healthcare settings. Patients with severe peripheral arterial ischemia, with diabetic foot gangrene, or requiring urgent revascularization or amputation may have been managed primarily in vascular surgery, endocrinology, or orthopedic departments, thereby contributing to referral and selection bias. Although the study included a relatively large number of geriatric patients with chronic lower extremity ulcers, several ulcer subtypes were represented by a limited number of cases, limiting etiology-specific interpretation. In addition, the majority of ulcers were superficial, whereas relatively few patients had muscle/tendon or bone involvement. Therefore, caution is warranted when extrapolating our findings to patients with more advanced or deeper ulcers. Furthermore, several variables contained missing data, and more than one-third of patients were lost to follow-up, which may have introduced selection bias and resulted in an underestimation of the overall healing rate. Missing data for some variables may also have influenced the precision of the regression analyses, particularly the Cox regression model. The retrospective design relied on the completeness and accuracy of routinely documented clinical data and precluded implementation of a uniform follow-up schedule. Follow-up intervals and duration therefore varied between patients, and patients with shorter observation periods may not have had sufficient time to reach the healing endpoint. This variability may have influenced the observed healing rates and the estimates obtained from the regression analyses. Finally, treatment decisions were not standardized but reflected routine clinical practice in a tertiary referral center. In addition, internal validation of the prediction models was not performed; therefore, their predictive performance should be interpreted with caution.

## 6. Conclusions

Chronic lower extremity ulcers in older adults represent a complex clinical problem characterized by a high burden of comorbidities, frequent microbiological involvement, and often prolonged healing courses. In this real-world study, peripheral arterial disease and greater ulcer depth were independently associated with poorer healing outcomes, while positive wound cultures emerged as a significant predictor of non-healing. Our results suggest that comprehensive assessment of vascular status and wound severity may help identify patients at increased risk of poor healing. Early identification of high-risk patients and a multidisciplinary management approach may help optimize healing outcomes and reduce the burden of chronic ulcers in the growing geriatric population.

## Figures and Tables

**Table 1:** Baseline Demographic, Clinical, and Ulcer Characteristics of the Study Population (*n* = 191).

	*n* (%)/Median (IQR)
**Demographics**	
Age, years	70 (65–76)
Male sex	111 (58.1)
BMI, kg/m^2^	27.4 (24.2–30.9)
**Comorbidities**	
Diabetes mellitus	114 (59.7)
Hypertension	108 (56.5)
Coronary artery disease/heart failure	67 (35.1)
Peripheral arterial disease	54 (28.3)
Chronic kidney disease	42 (22.0)
Malignancy	36 (18.8)
Immunosuppression	37 (19.4)
Atrial fibrillation	11 (5.8)
History of falls/trauma	39 (20.4)
Number of comorbidities, mean ± SD	2.5 ± 1.6
**Medications**	
Antiplatelet therapy	89 (46.6)
Anticoagulant therapy	35 (18.3)
**Smoking status** (*n* = 190)	
Never smoker	150 (78.9)
Former smoker	21 (11.1)
Current smoker	19 (10.0)
**Laboratory findings**	
CRP, mg/L	15 (4–48)
HbA1c, %	7.2 (6.1–8.2)
**Ulcer type** *	
Venous	71 (37.2)
Diabetic	37 (19.4)
Other/unclassified	26 (13.6)
Arterial	19 (9.9)
Mixed/multiple types	18 (9.4)
Vasculitic	13 (6.8)
Drug-induced	12 (6.3)
Pyoderma gangrenosum	7 (3.7)
Lymphatic	4 (2.1)
Neoplastic	2 (1.0)
**Ulcer characteristics**	
Number of ulcers	1 (1–2)
Duration, months	3 (1–8)
Area, cm^2^	4 (1–9)
**Location**	
Tibial region	86 (45.0)
Toe	40 (20.9)
Plantar heel/foot	23 (12.0)
Lateral malleolus	16 (8.4)
Dorsum of the foot	14 (7.3)
Medial malleolus	12 (6.3)
**Wound assessment**	
Superficial depth	116 (60.7)
Subcutaneous depth	63 (33.0)
Muscle/tendon involvement	10 (5.2)
Bone involvement	2 (1.0)
Necrosis	101 (53.2)
Fibrin	68 (35.6)
Pain	131 (68.9)
Malodor	73 (38.2)
**Exudate level**	
None	90 (47.1)
Mild	48 (25.1)
Moderate	29 (15.2)
Heavy	24 (12.6)

BMI, body mass index; CRP, C-reactive protein; HbA1c, glycated hemoglobin; SD, standard deviation; IQR, interquartile range. Categorical variables are presented as *n* (%), and continuous variables as median (IQR) unless otherwise specified. * Mixed ulcers were defined as ulcers with more than one underlying etiology.

**Table 2:** Microbiological and Imaging Findings (*n* = 191).

Variable	*n* (%)
**Culture results**	
Positive wound swab culture	92 (48.2)
Positive deep tissue culture	14 (7.3)
Positive blood culture	9 (4.7)
**Wound swab culture isolates**	
*Staphylococcus aureus* (overall)	32 (34.8)
MRSA	14 (15.2)
MSSA	18 (19.6)
*Pseudomonas* spp.	22 (23.9)
*Escherichia coli*	15 (16.3)
*Serratia* spp.	11 (12.0)
*Klebsiella* spp.	10 (10.9)
*Proteus* spp.	9 (9.8)
*Morganella* spp.	8 (8.7)
*Acinetobacter* spp.	6 (6.5)
*Enterobacter* spp.	5 (5.4)
*Enterococcus* spp.	5 (5.4)
**Wound swab culture characteristics**	
Gram-positive organisms	39 (42.4)
Gram-negative organisms	58 (63.0)
Polymicrobial growth	32 (34.8)
**Imaging findings**	
Abnormal arterial Doppler ultrasonography	45 (23.6)
Abnormal venous Doppler ultrasonography	36 (18.8)
MRI-confirmed osteomyelitis	22 (11.6)

MRSA, methicillin-resistant *Staphylococcus aureus*; MSSA, methicillin-susceptible *Staphylococcus aureus*; MRI, magnetic resonance imaging. Percentages for microbial isolates were calculated based on the number of positive wound swab cultures (*n* = 92). Data are presented as *n* (%).

**Table 3:** Treatment Modalities and Clinical Outcomes (*n* = 191).

Variable	*n* (%)/Median (IQR)
**Wound care interventions**	
Topical wound care	86 (45.0)
Negative-pressure wound therapy	142 (74.3)
Compression therapy	42 (22.0)
Debridement	46 (24.1)
Platelet-rich fibrin application	14 (7.3)
Boric acid dressing	50 (26.2)
**Antimicrobial therapy**	
Topical antibiotics (*n* = 188)	145 (77.1)
Systemic antibiotics	125 (65.4)
Duration of systemic antibiotic therapy, days (*n* = 127)	14 (10–28)
Antifungal therapy (topical/systemic)	65 (34.0)
**Surgical interventions**	
Revascularization procedures	11 (5.8)
Skin graft/flap surgery	2 (1.0)
Amputation	9 (4.7)
**Clinical outcomes** (*n* = 186)	
Complete healing	35 (18.8)
Partial healing	55 (29.6)
No healing	28 (15.1)
Lost to follow-up	68 (36.6)
**Follow-up and recurrence**	
Follow-up duration, months	2 (0–9)
Healing time, months (*n* = 85)	2 (1–6)
Any recurrence among healed patients (*n* = 90)	28 (31.1)
Time to first recurrence, months (*n* = 27)	8 (2–30)
**Hospitalization**	
Any hospitalization	67 (35.1)
Length of hospital stay, days (*n* = 66)	20 (11.2–48.8)

Outcome data were available for 186 patients. Missing outcome data were present in 5 patients. Healing time and hospitalization duration were calculated using available cases only.

**Table 4:** Multivariable Predictors of Healing Outcomes.

Variable	Complete Healing aOR (95% CI)	*p*-Value	Non-Healing aOR (95% CI)	*p*-Value	Time-to-Healing aHR (95% CI)	*p*-Value
Peripheral arterial disease	0.25 (0.09–0.64)	0.003	—	—	0.32 (0.13–0.79)	0.013
Ulcer depth (per grade increase)	0.45 (0.20–0.91)	0.026	2.36 (1.26–4.54)	0.007	0.49 (0.25–0.93)	0.030
Positive wound culture	0.62 (0.26–1.44)	0.264	5.12 (1.82–17.63)	0.001	0.68 (0.34–1.34)	0.261
	**Model Performance**					
	**Model**			**Performance Metric**		
	Complete healing model			AUC = 0.739		
	Non-healing model			AUC = 0.745		
	Time-to-healing model			C-index = 0.68		

aOR, adjusted odds ratio; aHR, adjusted hazard ratio; CI, confidence interval; AUC, area under the receiver operating characteristic curve. Complete healing and non-healing were evaluated using multivariable Firth penalized logistic regression models. Time-to-healing was evaluated using a multivariable Cox proportional hazards model. Predictor variables were selected a priori based on clinical relevance. Higher ulcer depth grades indicate deeper tissue involvement.

## Data Availability

The datasets generated and/or analyzed during the current study are not publicly available due to patient confidentiality and institutional restrictions but are available from the corresponding author on reasonable request.

## Supplementary Materials

The following supporting information can be downloaded at: https://www.mdpi.com/article/10.3390/jcm15156030/s1. Supplementary Table S1: Univariable screening of candidate predictors included in multivariable analyses.
