# Supplementary material for: Clinical Characteristics and Determinants of Healing in Older Adults with Chronic Lower Extremity Ulcers: A Retrospective Cohort Study
Source: J Clin Med. 2026 Aug 3;15(15):6030. doi: 10.3390/jcm15156030 (PMC13467550; doi:10.3390/jcm15156030)
Supplement: Supplementary file 1 [file jcm-15-06030-s001.zip › jcm-4434925-supplementary.pdf]

Supplementary Table S1. Univariable screening of candidate predictors included in multivariable analyses

Variable	Complete healing OR (95% CI)	p	Non-healing OR (95% CI)	p	Time to complete healing HR (95% CI)	p
Age	1.03 (0.98–1.08)	0.224	1.02 (0.97–1.08)	0.351	1.03 (0.99–1.07)	0.179
Female sex	1.34 (0.60–2.98)	0.470	0.76 (0.32–1.83)	0.541	1.17 (0.60–2.28)	0.639
Body mass index	0.91 (0.82–1.01)	0.069	0.98 (0.91–1.06)	0.649	0.93 (0.86–1.00)	0.061
Diabetes mellitus	0.67 (0.30–1.50)	0.331	1.20 (0.50–2.89)	0.685	0.65 (0.33–1.26)	0.203
Hypertension	0.93 (0.42–2.06)	0.849	1.38 (0.57–3.31)	0.476	0.84 (0.43–1.64)	0.612
Peripheral arterial disease	0.22 (0.08–0.59)	0.003	1.50 (0.64–3.53)	0.356	0.31 (0.13–0.75)	0.009
Coronary artery disease / heart failure	1.01 (0.45–2.26)	0.981	1.18 (0.50–2.79)	0.708	1.08 (0.55–2.12)	0.825
Chronic kidney disease	0.82 (0.32–2.08)	0.678	1.56 (0.61–3.95)	0.352	0.99 (0.45–2.19)	0.986
Malignancy	0.80 (0.29–2.25)	0.676	1.54 (0.56–4.24)	0.402	0.71 (0.30–1.72)	0.452
Immunosuppression	0.94 (0.33–2.66)	0.904	1.36 (0.47–3.93)	0.566	0.78 (0.32–1.88)	0.577
Comorbidity count	0.85 (0.67–1.08)	0.189	1.18 (0.92–1.52)	0.185	0.86 (0.70–1.06)	0.160
Ulcer duration	1.00 (0.98–1.01)	0.501	1.00 (0.99–1.01)	0.686	1.00 (0.98–1.01)	0.559
Ulcer area	1.00 (0.99–1.01)	0.397	1.00 (1.00–1.01)	0.245	1.00 (0.99–1.01)	0.503
Number of ulcers	0.98 (0.73–1.31)	0.891	1.18 (0.90–1.54)	0.244	0.96 (0.74–1.24)	0.762
Ulcer depth	0.41 (0.20–0.85)	0.016	2.57 (1.36–4.86)	0.004	0.47 (0.25–0.87)	0.017
Necrosis	0.98 (0.48–2.02)	0.957	1.11 (0.51–2.41)	0.787	0.88 (0.48–1.64)	0.692
Fibrin	0.65 (0.29–1.45)	0.294	2.46 (1.02–5.93)	0.044	0.76 (0.39–1.50)	0.433
Pain	1.05 (0.39–2.80)	0.928	0.87 (0.31–2.49)	0.802	1.05 (0.46–2.41)	0.904
Odour	0.66 (0.29–1.53)	0.332	3.42 (1.42–8.25)	0.006	0.79 (0.39–1.62)	0.528
C-reactive protein	1.00 (0.99–1.01)	0.380	1.01 (1.00–1.02)	0.007	1.00 (0.99–1.01)	0.460

HbA1c	0.77 (0.56–1.07)	0.125	1.16 (0.90–1.51)	0.256	0.85 (0.66–1.11)	0.237
Wound culture positive	0.48 (0.21–1.06)	0.070	6.00 (1.93–18.69)	0.002	0.55 (0.28–1.08)	0.082
Polymicrobial growth	0.48 (0.18–1.30)	0.149	2.26 (0.91–5.61)	0.077	0.64 (0.26–1.54)	0.315
MRI osteomyelitis	0.23 (0.05–1.06)	0.060	4.00 (1.42–11.27)	0.009	0.30 (0.07–1.27)	0.102
Arterial Doppler abnormal	0.57 (0.21–1.57)	0.279	2.22 (0.88–5.63)	0.092	0.60 (0.25–1.45)	0.256
Venous Doppler abnormal	0.53 (0.20–1.41)	0.204	1.83 (0.76–4.38)	0.176	0.59 (0.25–1.37)	0.220

OR, odds ratio; HR, hazard ratio; CI, confidence interval.
